# Plant-microbe interactions before drought influence plant physiological responses to subsequent severe drought

**DOI:** 10.1038/s41598-018-36971-3

**Published:** 2019-01-22

**Authors:** Danielle E. M. Ulrich, Sanna Sevanto, Max Ryan, Michaeline B. N. Albright, Renee B. Johansen, John M. Dunbar

**Affiliations:** 1 Bioscience Division, Los Alamos National Laboratory, Los Alamos, NM USA; 2 Earth and Environmental Sciences Division, Los Alamos National Laboratory, Los Alamos, NM USA

## Abstract

We examined the effect of soil microbial communities on plant physiological responses to drought. *Bouteloua gracilis* seeds were planted in sterilized sand with (inoculated) and without (controls) soil microbial communities. After substantial growth, drought was imposed by completely withholding water. Before soil moisture declined to zero, inoculated plants germinated faster, were significantly taller, and maintained greater soil moisture than controls. The greater soil moisture of the inoculated plants allowed greater photosynthesis but also induced lower tissue drought tolerance (as indicated by turgor loss point) compared to controls. The inoculated plants were more susceptible to severe drought compared to control plants as indicated by significantly lower mean stomatal conductance, as well as marginally significantly greater mean wilting score, for the entire severe drought period after soil moisture declined to zero. Inoculated plants exhibited enhanced growth and photosynthesis and dampened drought stress over short timescales, but also increased susceptibility to drought over long timescales. This work demonstrates (1) an unexpected insight that microbes can have positive initial effects on plant performance, but negative impacts on plant performance during severe drought, and (2) that microbially altered effects on plant function during well-watered and moderate drought conditions can influence plant function under subsequent severe drought.

## Introduction

Plants interact with diverse microorganisms including bacteria and fungi that can have positive, negative, or neutral effects on plants^1^. Positive effects arise from symbiotic relationships between plants and microbes where plants provide photosynthetically fixed carbon (e.g. root exudates, litter) to microbes, while in return microbes can increase abiotic stress tolerance, improve plant nutrient acquisition, promote growth, stimulate hormone production, and provide defense against pathogens^1–7^. Negative effects of microbes on plants include resource competition, parasitism, and pathogenesis that can reduce plant performance^1,3^.

Manipulation of plant-microbe interactions can improve plant performance in applications ranging from climate change mitigation to agricultural production. These manipulations can enhance different plant physiological metrics including productivity (i.e. yield, growth, photosynthesis), survival, stress tolerance, CO_2_ and nutrient uptake, and water use efficiency^1^. Thus, efforts have focused on redirecting the microbiome to enhance a plant function of interest during drought^8–15^. To modify the microbiome to enhance plant function, assemblages of beneficial microorganisms are introduced to host plants as plant species receive greater benefits from a more complex community than single strains^1,16–18^. Often, most of these efforts focus on one application (e.g. crop production, ecosystem restoration) and consequently enhance just one plant function such as growth or drought tolerance. However, improving whole plant performance via both growth and drought tolerance would enable plant-microbe interactions to benefit a broader suite of applications (agriculture, industry, bioenergy, ecosystem services).

To accomplish this, we need to improve our understanding of how the presence of soil microbial communities alters whole plant performance via both growth and drought tolerance in response to moderate and severe drought (i.e. zero soil moisture)^18,19^. Although microbial activity typically ceases after soil moisture declines to zero^20^, the effects of plants and soil microbes on each other and soil properties before soil moisture declines to zero may influence how plants respond to subsequent drought. For instance, soil microbes may produce plant hormones like abscisic acid or alter plant hormone production that can affect plant physiology related to growth and photosynthesis, and drought tolerance such as stomatal closure during drought^21–23^. Plants may use root exudates, a form of communication between plants and microbes^24^, to stimulate and recruit microorganisms with traits that benefit plants during a drought^1^. Soil microbes also secrete extracellular polysaccharides (EPS), glycoconjugates, and proteins that influence soil structure, soil water holding capacity, and nutrient transport^25–27^. These compounds released from both roots (e.g. exudates) and soil microbes (e.g. EPS) influence the rhizosphere and plant and microbial function during both well-watered and moderate drought conditions, which is expected to have cascading effects on plant physiological responses to subsequent drought after soil moisture declines to zero.

The goal of the present study was to evaluate how soil microbial communities influence whole plant performance via both growth and drought tolerance in response to moderate and severe drought in *Bouteloua gracilis*. *B. gracilis* is a native, perennial, warm season C4 grass that is highly drought resistant, allowing it to be a widespread and dominant species throughout semiarid grassland ecosystems in North America^28–30^. *B. gracilis* accounts for 75–90% of net primary productivity of grasslands^31,32^, regions expected to experience a greater intensity, frequency, and duration of drought throughout the 21^st^ century^33–35^. This will significantly impact these ecosystems because *B. gracilis* productivity (e.g. growth, photosynthesis) depends on the amount and distribution of rainfall^33,36^. *B. gracilis*’ relationship with soil microbes may contribute to its high drought resistance, nutrient acquisition, and wide geographic distribution^37^. However, the effect of soil microbial communities on *B. gracilis*’ physiology and capacity to inhabit water limited environments has not been investigated.

We examined two questions: (1) Given efforts to engineer beneficial plant-microbe interactions, do soil microbial communities improve both growth and drought tolerance in *B. gracilis*? and (2) Does the effect of soil microbial communities on *B. gracilis* function before drought influence plant physiological responses to subsequent severe drought? We hypothesized that (1) *B. gracilis* inoculated with soil microbial communities would exhibit both greater growth and greater drought tolerance compared to uninoculated controls, and (2) that plant-microbe interactions that occur during both well-watered and moderate drought conditions influence plant physiological responses to subsequent severe drought. We tested these hypotheses by growing *B. gracilis* in sterilized sand (controls) or sterilized sand inoculated with soil microbial communities. After 99 days of plant growth, drought was imposed on both groups by completely withholding water. Plant physiological measurements including growth (germination, height, biomass), photosynthesis, drought tolerance, stomatal conductance, and leaf wilting were made across the three periods of the experiment: well-watered (the period before drought began, days 1–98), moderate drought (the period after drought began before soil moisture declined to zero, days 99–117), and severe drought (the period after soil moisture declined to zero, days 118–131).

## Results

### Inoculated plants exhibited greater initial growth than controls

**Figure 1:**
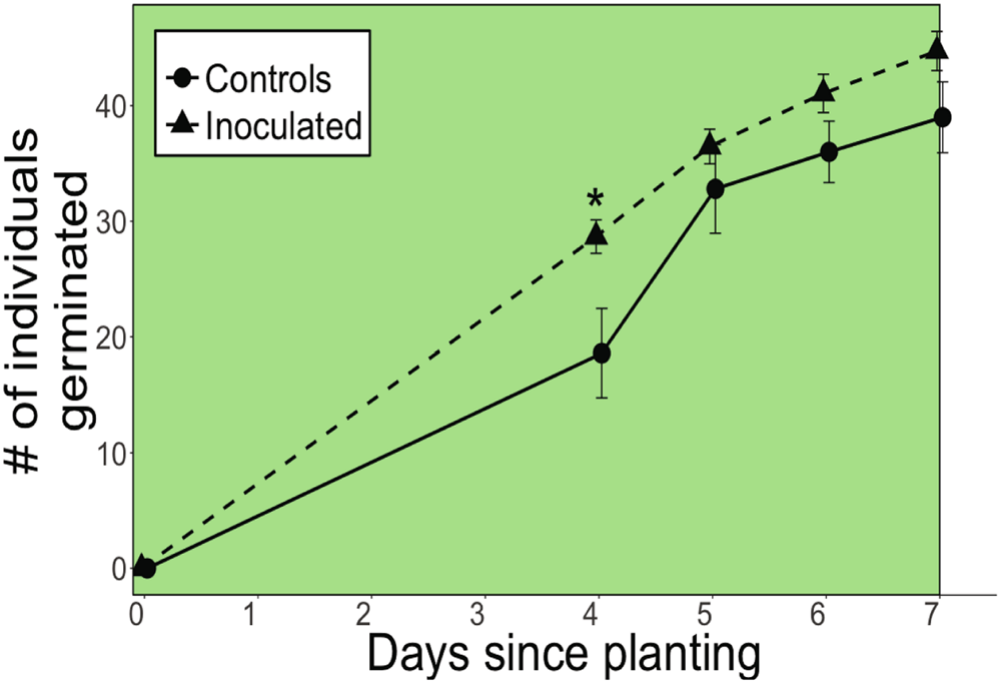
Number of individual plants that germinated for control and inoculated groups. Asterisks indicate significant differences between control and inoculated groups at P ≤ 0.05. All values are expressed as means ± SE. The green background color indicates the germination period during the well-watered period.

Inoculated plants exhibited consistently greater germination than controls and significantly greater germination than controls four days after planting (P ≤ 0.05, Fig. 1, Table 1). During days 4–7 of the well-watered period, mean number of individuals germinated of the inoculated group was significantly greater than that of controls (P = 0.024). Shoot height was also significantly greater in inoculated plants compared to controls 28 days after planting (P = 0.023, Table 2). Drought began on day 99 by completely withholding water. Root:shoot biomass ratio, root biomass, and shoot biomass measured on days 78 (before drought) and 132 (after drought), did not significantly differ between inoculated and control groups (P > 0.05, Supplementary Table S1). However, in both groups, root:shoot biomass ratio, root biomass, and shoot biomass after drought and after the experiment ended (day 132) were significantly lower than before drought (day 78) in both groups (P < 0.0001, Table 1, Supplementary Table S1).

**Table 1:** Summary of P-values from linear mixed effects models describing the effects of treatment, day, and the interaction (treatment x day) on germination, soil moisture, photosynthesis (*A*), stomatal conductance (*g*_s_), wilting score, and predawn leaf water potential.

	Germination	Root:shoot biomass	Soil moisture content	Photosynthesis (*A*)	Stomatal conductance (*g*_s_)	Wilting score	Predawn leaf water potential
Treatment	0.051	0.97	**0.014**	0.71	0.75	0.30	0.96
Day	**<0.0001**	**0.0009**	**<0.0001**	**<0.0001**	**<0.0001**	**<0.0001**	**<0.0001**
Treatment x Day	**0.020**	0.72	**<0.0001**	**0.01**	**0.03**	0.75	0.15

Significant values at P ≤ 0.05 are bolded.

**Table 2:** Physiological measurements of the control and inoculated groups during the well-waterd period before drought was imposed.

	Controls	Inoculated
Height (cm)	12.3 ± 0.40*	14.1 ± 0.25*
Ψ_TLP_ (bars)	−14.8 ± 0.61*	−12.8 ± 0.32*
*V*_cmax_	37.9 ± 9.1	44.7 ± 6.2
*J*_max_	34.8 ± 2.8	34.1 ± 1.4
*R*_d_	−0.0034 ± 0.5	−0.73 ± 0.3

Asterisks indicate statistically significant differences between controls and inoculated groups at P ≤ 0.05. All values are expressed as means ± SE. Turgor loss point (Ψ_TLP_), maximum rate of carboxylation (*V*_cmax_), maximum rate of electron transport (*J*_max_), and dark respiration (*R*_d_).

### Inoculated plants maintained greater soil moisture but were less drought tolerant than controls

**Figure 2:**
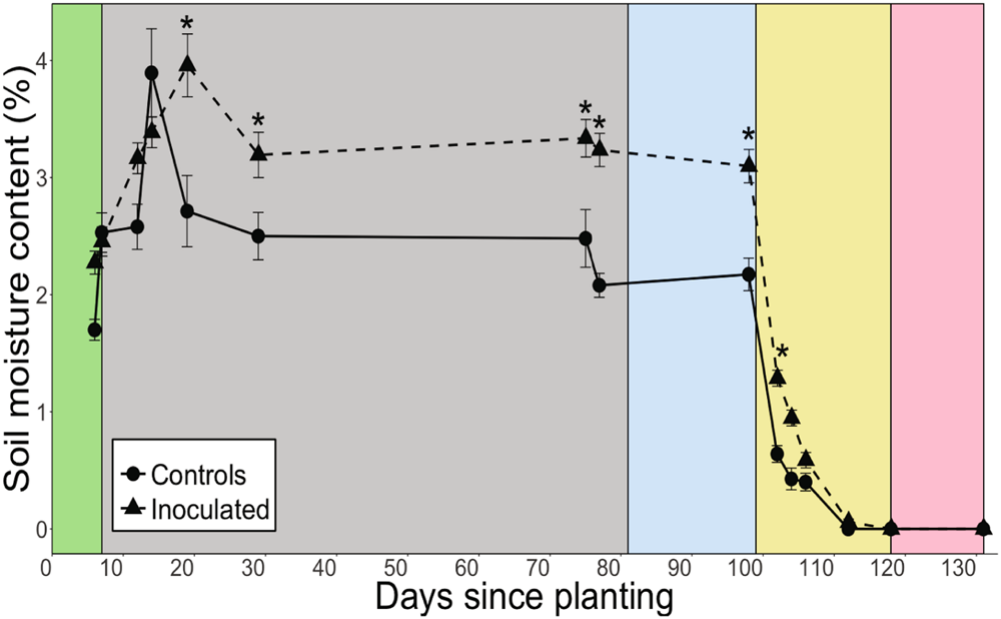
Soil moisture of control and inoculated groups. Asterisks indicate significant differences between control and inoculated groups at P ≤ 0.05. All values are expressed as means ± SE. The well-watered period includes the green, grey, and blue background colors (days 1–98; (green = germination, blue = gas exchange measurements), yellow indicates the moderate drought period before soil moisture declined to zero (days 99–117), and pink indicates the severe drought period after soil moisture declined to zero (days 118–131).

After germination, the inoculated group maintained significantly greater soil moisture than controls from day 19 through day 102 (P < 0.05, Fig. 2, Table 1) during the entire well-watered growth period and during moderate drought. The inoculated plants, however, exhibited lower drought tolerance than controls as indicated by the significantly less negative (i.e. greater) turgor loss point (Ψ_TLP_) of inoculated plants compared to controls (P = 0.017, Table 2). Prior to day 19, soil moisture did not significantly differ between the inoculated and control groups. Soil moisture of both the control and inoculated groups rapidly declined after drought began on day 99 until soil moisture of both groups declined to zero by day 118.

After the experiment ended on day 132, soil chemistry (C, C:N, Cu, Fe, K, Mn, N, NO_3_-N, organic matter, P, pH, Zn) and foliar N content did not significantly differ between groups (P > 0.05, Supplementary Table S2).

### Inoculated plants exhibited greater photosynthesis (*A*) before soil moisture declined to zero but lower stomatal conductance (*g*_s_) after soil moisture declined to zero

**Figure 3:**
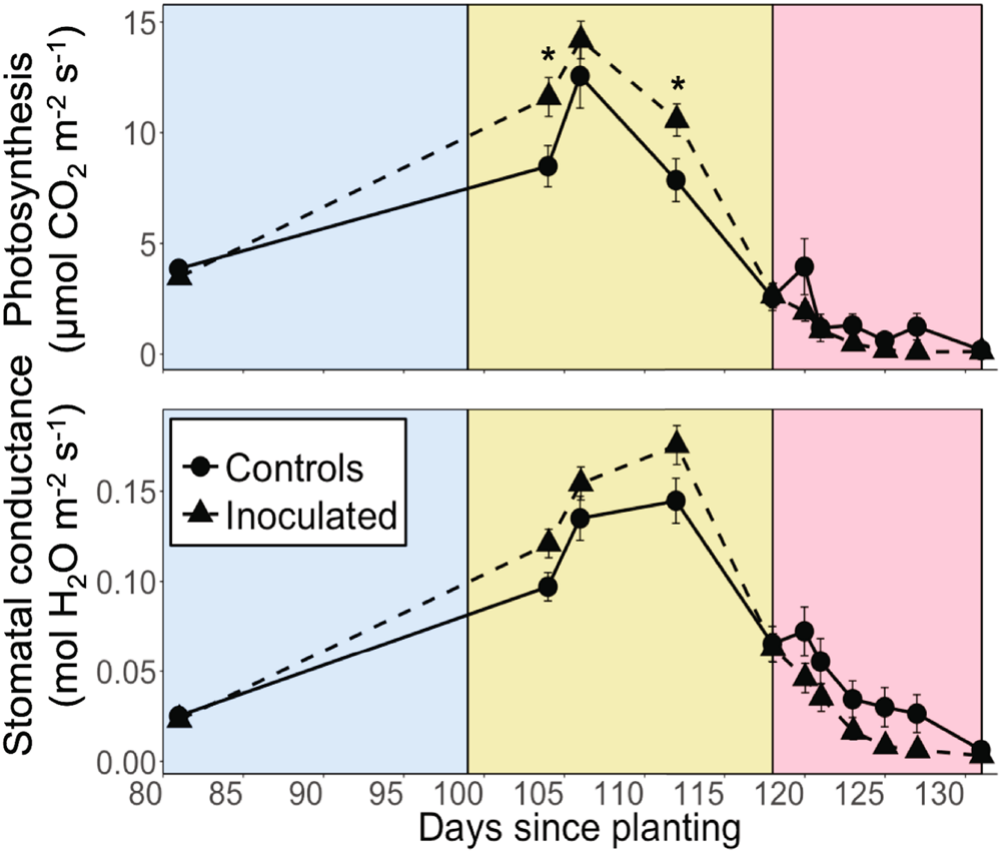
Photosynthesis and stomatal conductance of control and inoculated groups. Asterisks indicate significant differences between control and inoculated groups at P ≤ 0.05. All values are expressed as means ± SE. The blue background color indicates the well-watered period, yellow indicates the moderate drought period before soil moisture declined to zero (days 99–117), and pink indicates the severe drought period after soil moisture declined to zero (days 118–131). Mean photosynthesis of the inoculated group during the entire moderate drought period before soil moisture declined to zero (days 99–117, yellow background) was significantly greater than that of controls (P = 0.027). Mean stomatal conductance of the inoculated group during the entire severe drought period after soil moisture declined to zero (days 118–131, pink background) was significantly lower than that of controls (P = 0.042).

**Figure 4:**
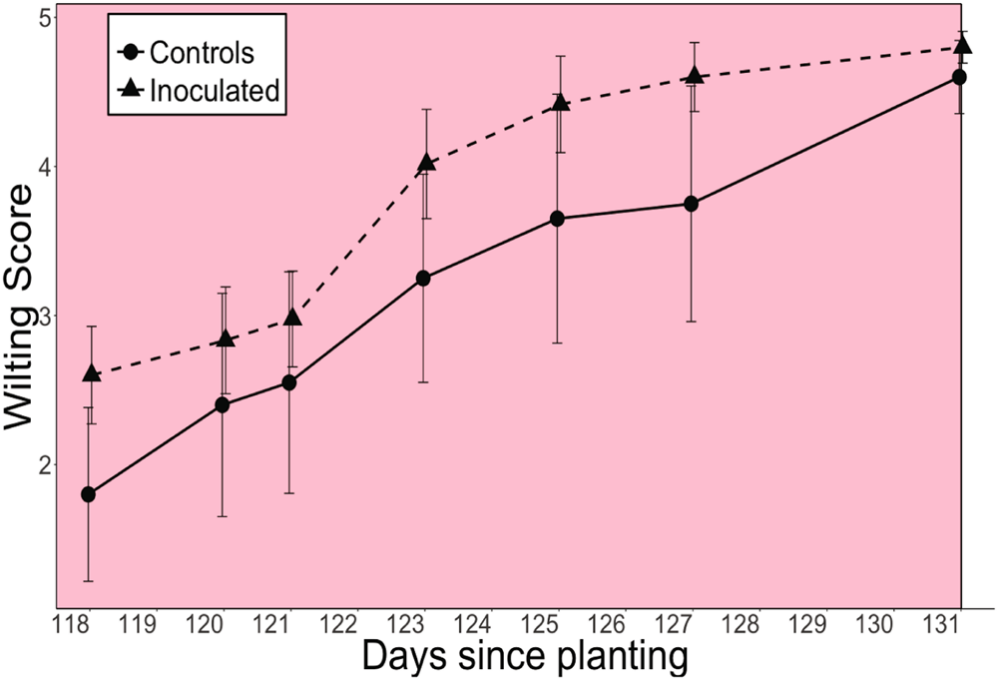
Wilting score of control and inoculated groups. All values are expressed as means ± SE. The pink background color indicates the severe drought period after soil moisture declined to zero (days 118–131). Mean wilting score of the inoculated group during the entire severe drought period after soil moisture declined to zero (days 118–131, pink background) was marginally significantly greater than that of controls (P = 0.058).

*A* of the inoculated group was significantly greater than that of controls during moderate drought on days 104 and 112 (P = 0.014, 0.030), and mean *A* of the inoculated group during the entire moderate drought period before soil moisture declined to zero (days 99–117, yellow background, Fig. 3) was significantly greater than that of controls (P = 0.027). After day 118, mean *g*_s_ of the inoculated group during the entire severe drought period after soil moisture declined to zero (days 118–131, pink background, Fig. 3) was significantly lower than that of controls (P = 0.042). The day 118 turning point coincided with the day at which soil moisture of both inoculated and control groups declined to zero and drought became severe (Fig. 2). Thus, control plants maintained stomatal opening longer than inoculated plants during severe drought. Mean wilting score of the inoculated group during the entire severe drought period (days 118–131, pink background, Figs 3 and 4) was marginally significantly greater than that of controls (P = 0.058, Fig. 4). Table 1 contains the results from the linear mixed effects models for *A*, *g*_s_, and wilting score.

Photosynthetic biochemical parameters describing photosynthetic efficiency–maximum rate of carboxylation (*V*_cmax_), maximum rate of electron transport (*J*_max_), and dark respiration (*R*_d_)–measured before drought did not significantly differ between inoculated and control groups (P = 0.60, P = 0.87, P = 0.32, respectively, Table 2). Predawn water potential did not significantly differ between inoculated and control groups (P = 0.96, Supplementary Fig. S1, Table 1).

## Discussion

Contrary to our first hypothesis that soil microbes would positively influence both growth and drought tolerance *of B. gracilis*, we found that soil microbial communities can elicit a mixture of positive and negative effects on plant physiological responses to drought, depending on the plant metric of interest. This also contrasts with previous research that reports either positive or negative effects of microbes and focus on only one plant function^1,38–40^. Drought began on day 99 by completely withholding water from inoculated and control *B. gracilis* and we observed a time- and drought-dependent tradeoff where soil microbial communities dampened drought stress in the short term during the moderate drought period before soil moisture declined to zero (days 99–117) but increased drought susceptibility in the long-term to severe drought after soil moisture declined to zero (days 118–131) during the severe drought period. In terms of drought susceptibility, inoculated plants functionally shut down (as indicated by stomatal conductance (*g*_s_), Fig. 3) and exhibited consistently greater wilting (Fig. 4) than controls during the severe drought period (Fig. 2). However, inoculated plants outperformed controls in terms of germination, height, and photosynthesis (*A*) during the well-watered and moderate drought periods before soil moisture had completely declined to zero. Thus, soil microbial communities had both positive (increased growth and photosynthesis) and negative (increased drought susceptibility) effects on plant physiological response to drought, an overlooked outcome of plant-microbe interactions. This finding impacts efforts to engineer beneficial plant-microbe interactions, highlighting the need to specify the target outcome and time-scale at which the positive/negative influence is evaluated.

Consistent with our second hypothesis, our results revealed that the effects of soil microbial communities on plant function during well-watered and moderate drought conditions can greatly impact plant physiological responses to subsequent severe drought (i.e. zero soil moisture). Soil microbes alter plant function through various mechanisms including modulating nutrient availability and uptake, and soil moisture^1,5^. In our study, the microbially-enhanced growth-drought susceptibility tradeoff was modulated by soil moisture content rather than nutrient availability because soil chemistry and foliar N content (Supplementary Table S2) did not significantly differ between groups. Our findings also indicate that the presence of soil microbial communities during the well-watered and moderate drought periods substantially influenced soil moisture and subsequent plant physiological responses to severe drought. The greater soil moisture of inoculated *B. gracilis* compared to controls for the majority of the experiment (days 19–102) dampened the effect of the drought on the inoculated plants because it delayed the onset of severe drought stress (i.e. zero soil moisture; Fig. 2); however, it also allowed inoculated plants to develop less drought tolerant tissues than controls as indicated by the Ψ_TLP_ (Table 2). The lower drought tolerance made inoculated plants more susceptible to drought when zero soil moisture finally did occur on day 118, as indicated by lower *g*_s_ and greater wilting compared to controls during severe drought^41,42^. Consequently, soil microbial communities promoted the opportunistic growth pattern of *B. gracilis* by enhancing growth (as indicated by germination, height) and *A* when soil moisture was available but also by encouraging greater functional inactivity as indicated by significantly lower mean *g*_s_ and marginally significantly greater mean wilting during the entire severe drought period after soil moisture declined to zero (Fig. 3). This enhanced tradeoff between drought susceptibility and growth is a tenet of plant ecophysiology^43,44^ that emphasizes the role that soil microbial communities may play in influencing plant growth patterns and the capacity of *B. gracilis* to thrive in drought-prone regions.

The inoculated group may have maintained greater soil moisture than controls because soil microbes can modify soil structure, influencing available water and nutrients^45^. For example, microbes secrete EPS that can hold several times its weight in water^46^ and thus increase water retention^27^ and promote soil aggregation^26^. ^46^found that sand amended with EPS held 100–500% more water and dried significantly slower than sand without EPS during just 14 hours of drying. Sand is also a fast-draining soil type, suggesting that the effect of soil microbial communities and EPS on soil moisture may have been particularly pronounced in this study. Soil microbes can also produce plant hormones that can alter plant physiology and soil water use such as stomatal closure due to microbially-produced abscisic acid^21–23^. The inoculated group may have also exhibited greater soil moisture than controls because of increased production of root exudates (ions, free oxygen and water, enzymes, mucilage, carbon-containing primary and secondary metabolites^3^) that stabilize soil structure^47^ and increase soil water-holding capacity^24,48^. Enhanced root growth can also improve water uptake^49^ and may improve soil water holding capacity^47^. However, we found no significant differences in root biomass nor root:shoot biomass ratios between groups before or after drought (days 78, 132, respectively, Supplementary Table S1). Although root biomass did not differ between groups, increased root branching could lead to increases in root surface area without increases in total biomass^50^. In response to drought, root:shoot biomass ratios often increase because plants stimulate root growth at the expense of shoot growth to access deeper water sources. However, it is likely that root mortality contributed to the reduced root:shoot biomass ratio observed in this study because root:shoot biomass measurements were taken at the end of the experiment after the plants had died.

The microbially-enhanced soil moisture of the inoculated group during well-watered and moderate drought conditions (days 19–102) led to reduced plant tissue drought tolerance, as measured by Ψ_TLP_ , which had cascading impacts on plant gas exchange during the severe drought period after soil moisture eventually declined to zero (days 118–131). This is because Ψ_TLP_ is a plant functional trait that represents the leaf water potential that induces wilting. Consequently, Ψ_TLP_ strongly underlies and is predictive of plant drought tolerance and species distributions relative to water availability in diverse ecosystems^51–54^. Species with a lower (more negative) Ψ_TLP_ are more able to withstand leaf dehydration, allowing the plant to maintain photosynthesis, stomatal conductance, and growth under drier conditions^55,56^. The effect of the reduced drought tolerance, as measured by Ψ_TLP_ , on the inoculated group’s increased drought susceptibility after soil moisture declined to zero highlights the role that soil microbial communities (Supplementary Fig. S2) may play early in *B. gracilis*’ life cycle. The results suggest that soil microbial communities can direct plant responses to eventual severe drought because they can influence soil moisture conditions during well-watered and moderate drought conditions.

The greater height of inoculated plants at day 28 (Table 2) suggests that soil microbial communities may stimulate fast growth early in the life cycle of *B. gracilis* and when soil moisture is available, consistent with the significantly greater germination of inoculated plants compared to controls (Fig. 1) and *B. gracilis*’ opportunistic growth strategy^36^. At day 78 when all plants had reached maturity, shoot biomass was only 15% greater in inoculated than control plants (Supplementary Table S1), suggesting that the early stimulatory growth effect may have been reduced when plants reached maturity. Nonetheless, during days 81–112 before soil moisture declined to zero, *A* of inoculated plants was 21% greater than controls (Fig. 3). This suggests that even when plants reached maturity, the inoculation of soil microbial communities positively influenced mature plant *A* before soil moisture declined to zero.

Soil microbes did not appear to influence photosynthetic biochemistry as indicated by *V*_cmax_, *J*_max_, and *R*_d_ (Table 2) although instantaneous measures of *A* were greater in inoculated plants than controls before soil moisture declined to zero (Fig. 3). The greater photosynthetic rates without changes in biochemistry may be due to a lower resistance to gas and liquid phase diffusion as observed in *B. gracilis* inoculated with its mycorrhizal symbiont *Glomus fasciculatus*^50^.

In this study, both *A* and *g*_s_ track the same trajectory (i.e. consistently greater *A* and *g*_s_ of inoculated plants during moderate drought, consistently lower *A* and *g*_s_ of inoculated plants during severe drought). However, the difference between groups during moderate drought was statistically significant for *A* but not *g*_s_, and similarly, the difference between groups during severe drought was statistically significant for *g*_s_ but not *A*. During moderate drought, this discrepancy between *A* and *g*_s_ in statistically significant treatment differences may occur because inoculated plants exhibited an 18% greater *V*_cmax_ than controls (Table 2), promoting the significantly greater *A* and only consistently greater *g*_s_ of inoculated plants compared to controls. During severe drought, this discrepancy may occur because non-stomatal limitations such as boundary layer and mesophyll conductances may play a larger role than stomatal limitations in limiting *A* at low *g*_s_ (<0.05 mol m^−2^ s^−1^)^42^. These non-stomatal limitations at low *g*_s_ outweigh the effect of the statistically significant *g*_s_ differences between groups on *A*.

This work provides unexpected insights into plant-microbial interactions: soil microbial communities can simultaneously have positive and negative impacts on plant performance depending on the plant metric of interest, and the presence of soil microbial communities early in *B. gracilis*’ life cycle can influence plant physiological responses to eventual severe drought. Soil microbial communities enhanced plant growth and photosynthesis and dampened drought stress over short timescales before soil moisture declined to zero, but increased plant susceptibility to drought over long timescales after soil moisture declined to zero. The tradeoff between growth and susceptibility to drought was modulated by soil moisture conditions before the onset of severe drought and demonstrated how soil microbial communities promoted the opportunistic growth pattern of *B. gracilis*. This work advances the engineering of beneficial plant-microbe interactions and our understanding of how soil microbial communities influence plant physiological responses to drought.

## Methods

### Plant material

*B. gracilis* is a warm season C4 fast growing grass typical to arid areas in Southwestern USA. It is highly responsive to variation in precipitation^33^ and exhibits an opportunistic growth pattern where it grows fast when water is available to take advantage of favorable growing conditions^36^ and dies back and becomes dormant when water is scarce^57,58^. In the field, *B. gracilis* is colonized by diverse microbes including mycorrhizal, dark septate fungi, endophytic, coprophilous, saprophytic, and plant pathogenic fungi that influence its physiology^37,59,60^. Seed was obtained from a nursery in Santa Fe, New Mexico, USA. Plants were grown under a 14-h photoperiod in a temperature-controlled greenhouse at the New Mexico Consortium in Los Alamos, New Mexico, USA. Seeds were planted 1 June 2017 (day 0 of experiment) and the experiment ended 10 October 2017 (day 131). During this period, average daytime temperature in the greenhouse was 22.6 °C, average nighttime temperature 20.6 °C, average daytime relative humidity 47.5%, and average daily maximum photosynthetic photon flux density (PPFD) was 382.4 umol m^−2^ s^−1^.

### Experimental Setup

Inoculated plants were inoculated with soil microbial communities from 15 geographically distinct New Mexico soils collected 0–5 cm below the surface (Supplementary Table S3) with distinct bacterial and fungal community composition (Supplementary Fig. S2). To create soil inocula, microbes from each soil were extracted by suspension in a phosphate buffered solution (PBS) to create a 1:20 dilution of each microbial suspension; this isolates the microbial community and eliminates any potential biogeochemical effects of the original soil. Aliquots of the soil inocula were set aside for DNA sequencing (see below). Before planting, seeds were surface sterilized for 10 min in 10% bleach and rinsed for 15 min in sterile DI twice. Seeds were then soaked in soil inocula (inoculated) or PBS (controls) for 10 min. Seeds were planted in 9.6 L pots of sterilized sand. Ten seeds were planted in each of 15 positions (wells) evenly spaced across each pot. Five mL of soil inocula (inoculated) or PBS (controls) was applied to each well once during initial planting and also a second time 11 days after planting to ensure effects of soil microbial communities (e.g.^61^). The soil inocula treatment was applied to 15 pots that formed the inoculated group (N = 15) where each pot was inoculated with a different community. The only-PBS treatment was applied to five pots that formed the control group (N = 5). Clear plastic covers were placed over all 20 pots to maintain high humidity to promote germination for 11 days after planting. A fertilizer treatment of ammonium nitrate (1 mg/mL) was applied evenly to all pots 15 days after planting. Plants were eventually thinned to 15 individuals per pot (1 individual per position) 30 days after planting. All 20 pots were well-watered every other day to field capacity using RO water sterilized with a 0.2 µm filter. Pot position was rotated biweekly. After substantial growth (99 days after planting), drought was imposed on both groups by completely withholding water. Both groups were droughted until death (complete desiccation). Because we completely withheld water to impose drought until plants died, drought severity increased with time. The period before drought began is considered well-watered conditions (days 1–98), the period after drought began before soil moisture declined to zero is considered moderate drought (days 99–117), and the period after soil moisture declined to zero is considered severe drought (days 118–131). Plants did not turn to seed during the experiment. Because all plants were planted at the same time, and differences in germination and growth rates were relatively small, phenology did not influence the results.

### Measurements

Germination was determined by counting the total number of individual shoots in each pot 4–7 days after planting. The height of the tallest tiller in each position was measured prior to maturity 28 days after planting (before pots were thinned) for a total of 15 heights per pot.

Photosynthesis (*A*) and stomatal conductance (*g*_s_) were measured using a portable photosynthesis system with an infrared gas analyzer (LI-6400 XT, Licor, Lincoln, NE, USA) before drought and then periodically throughout the drought until plants died. In the cuvette, flow rate was set to 500 μmol s^−1^, reference [CO_2_] 400 μmol mol^−1^, quantum flux 2000 μmol m^−2^ s^−1^ to avoid any light limitation of photosynthesis, and leaf temperature 20 °C. Leaf area was used to normalize gas exchange values. Gas exchange was measured on the middle sections of 2 leaves, resulting in two rectangles of leaf material contained in the cuvette. This allowed leaf area to be determined by measuring the widths of each of the 2 leaf rectangles. Widths were multiplied by 3 cm (length of the cuvette) and summed to calculate total leaf area in the chamber. Middle sections of leaves were marked so the same leaves could be measured over time and to avoid destructive sampling.

Soil moisture was measured with a handheld volumetric water content soil moisture sensor (WaterScout SM 100, Spectrum Technologies, Aurora, IL, USA) to a depth of 6 cm (half the height of the sand in each pot) at 3 different positions in each pot weekly before (on the days between watering), during, and after drought.

Predawn leaf water potential measurements were taken on 3 leaves per pot before and throughout the drought approximately once per week using a pressure chamber (PMS Instrument Company, Albany, OR, USA). Shoots were sampled at predawn, sealed in ziplock bags with a moist paper towel, and stored at 4 °C until measurement. Measurements were made within 30 min of sampling.

Photosynthetic biochemical parameters were determined from photosynthesis-[CO_2_] (*A*-*C*_i_) curves measured before drought on 2 leaves (using the same cuvette conditions as described above) at a range of CO_2_ concentrations (μmol mol^−1^) of: 400, 300, 200, 100, 50, 400, 600, 800, 1000, 1200, 1400. Maximum rate of carboxylation (*V*_cmax_), maximum rate of electron transport (*J*_max_), and dark respiration (*R*_d_) were determined using the ‘plantecophys’ R package that uses non-linear regression to fit the *A*-*C*i curve^62^. *A*-*C*i curves were measured on 3 plants per pot over 9 days (days 89–97) before drought.

The water potential at turgor loss or turgor loss point (Ψ_TLP_) was determined from pressure-volume (*P*-*V*) curves as in^50^. Ψ_TLP_ is a measure of drought tolerance^51^. Species with lower (more negative) Ψ_TLP_ are more tolerant of drought^55,56^. *P*-*V* curves were measured on 3 plants per pot (60 curves total) before drought over 29 days (days 43–71). Shoots were sampled at predawn and were then rehydrated for 2–3 hours. No rehydration-induced plateau was detected^63^. Shoots were allowed to dry out slowly on the laboratory bench. Measurements of shoot mass and balance pressure taken with the pressure chamber were recorded as shoots dried out. Data were plotted with relative water deficit on the x-axis and 1/Ψ on the y-axis. Data were checked during measurement to ensure at least 3–5 points were recorded along the linear portion of the curve. Ψ_TLP_ was estimated from the intersection of the linear portion of the curve with a negative exponential function fitted to the non-linear portion. To test if rehydration affected *P-V* curves^64^, two individuals from each of five randomly selected pots were sampled; one individual was rehydrated for 2–3 hours and one was not rehydrated. *P-V* curves were measured on the rehydrated and non-rehydrated plants. Ψ_TLP_ of rehydrated (−1.62 + 0.24 MPa) and non-rehydrated (−1.55 + 0.11 MPa) plants did not significantly differ (P = 0.82, N = 5).

After soil moisture declined to zero (day 118), wilting score was visually determined on a scale from 1–5: green, no leaf curling/rolling (1); green, some leaf curling (2), green, leaf curling (3), green, dried out (4), brown, completely desiccated, dead (5) (e.g.^65^).

Three plants per pot were sampled for biomass of roots and shoots before drought (day 78) and after the experiment ended (day 132). All roots were sampled. Roots and shoots were dried at 60 °C and the mass of each tissue was recorded. We also measured foliar N content (%) of shoots collected on day 78. To check roots for arbuscular mycorrhizal fungal infection, roots were cleared with 5% KOH, acidified in 5% HCl, and stained in a 1:1:1 mix of DI H_2_O, lactic acid, glycerol, and 0.05% trypan blue (protocol adapted from^66^). Roots were visually assessed for colonization using light microscopy. Analysis of stained roots indicated minimal arbuscular mycorrhizal fungal infection in both control and inoculated groups.

At the end of the drought, the soil chemical composition (C, C:N, Cu, Fe, K, Mn, N, NO_3_-N, organic matter, P, pH, Zn) of a subset of pots (N = 4 controls, N = 8 inoculated) was measured at the Colorado State University Soil, Water, and Plant Testing Laboratory.

### DNA extraction and sequence processing and analysis

DNA extractions were performed using the DNeasy PowerSoil DNA extraction kit (Qiagen). The standard protocol was used: (1) 0.3 grams of material was used per extraction; (2) bead beating was conducted using a Spex Certiprep 2000 Geno/Grinder for three minutes at 1900 strokes/minute. DNA samples were quantified with an Invitrogen Quant-iT^TM^ ds DNA Assay Kit on a BioTek Synergy HI Hybrid Reader. PCR templates were prepared by diluting an aliquot of each DNA stock in sterile water to 1 ng/µl. The bacterial (and archaeal) 16 S rRNA gene (V3-V4 region) was amplified using primers 515f-R806^67^. Henceforth, archaeal sequences were analysed with bacterial sequences. The fungal 28 S rRNA gene (D2 hypervariable region) was amplified using the LR22R primer^68^ and the reverse LR3 primer^69^. Preparation for Illumina high-throughput sequencing was undertaken using a two-step approach, similar to that performed by^70^, with Phusion Hot Start II High Fidelity DNA polymerase (Thermo Scientific). In the first PCR, unique 6 bp barcodes were inserted into the forward and reverse primer in a combinatorial approach over 22 cycles with an annealing temperature of 60 °C^71^. The second PCR added Illumina-specific sequences over 10 cycles with an annealing temperature of 65 °C. Amplicons were cleaned using a Mobio UltraClean PCR clean-up kit, quantified using the same procedure as for the extracted DNA, and then pooled at a concentration of 10 ng each. All clean ups were undertaken as per the manufacturer’s instructions with the following modifications: binding buffer amount was reduced from 5X to 3X sample volume, and final elutions were performed in 50 µl Elution Buffer. A bioanalyzer was used to assess DNA quality, concentration was verified using qPCR, and paired-end 250 bp reads were obtained using an Illumina MiSeq sequencer at Los Alamos National Laboratory.

Bacterial and fungal sequences were merged with PEAR v 9.6^72^, quality filtered to remove sequences with 1% or more low-quality (q20) bases, and demultiplexed using QIIME^73^ allowing no mistmatches to the barcode or primer sequence. Further processing was undertaken with UPARSE^74^. Sequences with an error rate greater than 0.5 were removed, remaining sequences were dereplicated, singletons were excluded from clustering, OTU clustering was performed at 97% and putative chimeras were identified *de novo* using UCHIME^75^. Bacterial and fungal OTUs were classified using the Ribosomal Database Project (RDP) classifier^76^. The OTUs which were not classified as bacteria or fungi with 100% confidence were removed from the dataset. Bacterial OTUs also had to have a phylum classification confidence level of at least 80% to remain in the dataset.

Community composition analyses were performed with rarefied data unless otherwise stated. Bacterial communities were rarefied to 756 sequences per sample. Fungal communities were rarefied to 2333 sequences. Bray-Curtis dissimilarity matrices for bacterial and fungal communities were computed for bacteria and fungi. To test that all 15 soil microbial communities were compositionally distinct, we evaluated the homogeneity of multivariate dispersions. We found that the average distance to the median was 0.4781 for bacteria and 0.6038 for fungi and visualized soil microbial community differences using non-metric multidimensional scaling plots (vegan v 2.4–3^77^).

### Statistical analyses

Linear mixed effects models were used to determine differences in response variables between inoculated and control groups. Fixed effects were treatment (inoculated, control) and day, the random effect was pot, and response variables were germination, soil moisture, *A*, *g*_s_, root:shoot biomass ratio, predawn leaf water potential, and wilting score (Table 1). To account for repeated measurements through time, models with different correlation structures were fit. The model of best fit was selected based on Akaike information criterion (AIC) values. Assumptions of constant variance and normality were checked using residual and quantile-quantile plots. All interactive and main effects of factors on the response were tested using marginal F-tests (also known as type III tests) that account for unbalanced sample sizes. To determine treatment differences in mean *A*, *g*_s_, and wilting score for each of the three periods of the experiment (well-watered (days 1–98), moderate drought (days 99–117), severe drought (days 118–131)), we included period as a fixed effect in the linear mixed effects models as well. Post-hoc comparisons were made using a 95% confidence interval and P ≤ 0.05. Welch’s two-sample t-tests that account for unbalanced sample sizes compared mean differences in shoot height, Ψ_TLP_ , *V*_cmax_, *J*_max_, *R*_d_, and soil chemistry between inoculated and control groups. All statistical analyses were conducted in R version 3.4.2^78^.

## Supplementary information

Supplementary Information
