# Supplementary material for: Plant-microbe interactions before drought influence plant physiological responses to subsequent severe drought
Source: Sci Rep. 2019 Jan 22;9:249. doi: 10.1038/s41598-018-36971-3 (PMC6342978; doi:10.1038/s41598-018-36971-3)
Supplement: Supplementary file 1 — Supplementary Information [file 41598_2018_36971_MOESM1_ESM.docx]

**SUPPLEMENTARY INFORMATION**

Title: Plant-microbe interactions before drought influence plant physiological responses to subsequent severe drought

Authors: Danielle E. Marias^1^, Sanna Sevanto^2^, Max Ryan^2^, Michaeline B. N. Albright^1^, Renee B. Johansen^1^, John M. Dunbar^1^

^1^Bioscience Division, Los Alamos National Laboratory, Los Alamos, NM, USA

^2^Earth and Environmental Sciences Division, Los Alamos National Laboratory, Los Alamos, NM, USA

Corresponding author:

Danielle E. Marias

P.O. Box 1663 MS M888

Los Alamos National Laboratory

Los Alamos, NM 87545 USA

505-667-8161

[daniellem@lanl.gov](mailto:daniellem@lanl.gov)

**Table S1.** Root biomass, shoot biomass, and root:shoot ratio of control (N=5) and inoculated (N=15) groups before drought (day 78) and after drought (day 132) began. Significant differences were detected between before and after drought periods but not between control and inoculated groups (Welch’s two sample t-test, P≤0.05).

|  | | Controls | Inoculated |
| --- | --- | --- | --- |
| Before drought  (day 78) | Root (g) | 0.085 ± 0.01 Aa | 0.098 ± 0.01 Aa |
|  | Shoot (g) | 0.053 ± 0.01 Aa | 0.061 ± 0.002 Aa |
|  | Root:Shoot | 1.41 ± 0.2 Aa | 1.59 ± 0.09 Aa |
| After drought  (day 132) | Root (g) | 0.15 ± 0.02 Ba | 0.13 ± 0.01 Ba |
|  | Shoot (g) | 0.23 ± 0.03 Ba | 0.27 ± 0.02 Ba |
|  | Root:Shoot | 0.69 ± 0.1 Ba | 0.60 ± 0.07 Ba |

^Uppercase letters indicate significant differences between before drought and after drought periods at P≤0.05.^

^Lowercase letters indicate significant differences between control and inoculated groups at P≤0.05.^

^All values are expressed as means ± SE.^

**Table S2.** Soil chemistry and foliar N content of control and inoculated groups. No significant differences between groups were observed (Welch’s two sample t-test, P>0.05).

|  | **Controls** | **Inoculated** |
| --- | --- | --- |
| **C** (%) | 0.110 ± 0.0032 | 0.102 ± 0.0061 |
| **C:N** | 7.65 ± 1.7 | 5.62 ± 0.71 |
| **Cu** (ppm) | 0.313 ± 0.086 | 0.299 ± 0.044 |
| **Fe** (ppm) | 0.798 ± 0.071 | 0.813 ± 0.032 |
| **K** (ppm) | 1.85 ± 0.26 | 3.65 ± 1.2 |
| **Mn** (ppm) | 0.748 ± 0.093 | 0.846 ± 0.046 |
| **N** (%) | 0.0165 ± 0.0032 | 0.0197 ± 0.0022 |
| **NO_3_-N** (ppm) | 0.198 ± 0.10 | 0.191 ± 0.049 |
| **Organic matter** (%) | 0.1 ± 0.0 | 0.1 ± 0.0 |
| **P** (ppm) | 7.40 ± 1.1 | 8.96 ± 0.70 |
| **pH** | 6.70 ± 0.27 | 6.50 ± 0.17 |
| **Zn** (ppm) | <0.01 | <0.01 |
|  |  |  |
| **Foliar N (%)** | 0.68 ± 0.03 | 0.65 ± 0.04 |

**Table S3.** Soil microbial communities were extracted from 15 geographically distinct soils collected in New Mexico in spring of 2016.

| Soil # | Longitude | Latitude | Elevation (m) | General plant cover |
| --- | --- | --- | --- | --- |
| 1 | -103.0675333 | 35.18281667 | 3810 | Grass, cover 100%, clumps ~20%. 1 inch of litter. |
| 2 | -106.099895 | 36.006518 |  | arid grass/juniper |
| 3 | -106.2276833 | 35.51945 | 5663 | grass prairie, bunchgrass |
| 4 | -106.7975167 | 35.07991667 | 5713 | grass prairie, tumbleweed pile |
| 5 | -106.9493 | 35.03866667 | 5273 | grass prairie, bunchgrass |
| 6 | -107.5607167 | 35.06951667 | 6116 | arid grassland/juniper, bunchgrass |
| 7 | -107.9893 | 34.9701 | 7211 | pine/grass woodland, grass/pinestraw |
| 8 | -108.1351667 | 35.01253333 | 7669 | pine/grass woodland |
| 9 | -108.6797333 | 35.11636667 | 6637 | arid grassland/juniper, shrub |
| 10 | -108.9344667 | 35.05485 | 6245 | juniper woodland |
| 11 | -108.2593667 | 36.73798333 | 5394 | grass/shrubland, bush |
| 12 | -107.9965333 | 36.65713333 | 5629 | grass/juniper woodland, juniper |
| 13 | -107.1373 | 36.07496667 | 7343 | pine/grass woodland, pine & bunchgrass |
| 14 | -106.8638167 | 35.57101667 | 5649 | grass/shrubland, sage-like plant |
| 15 | -10.98381667 | 35.71195 | 8225 | shrub/grassland; sage |

**FIGURES**


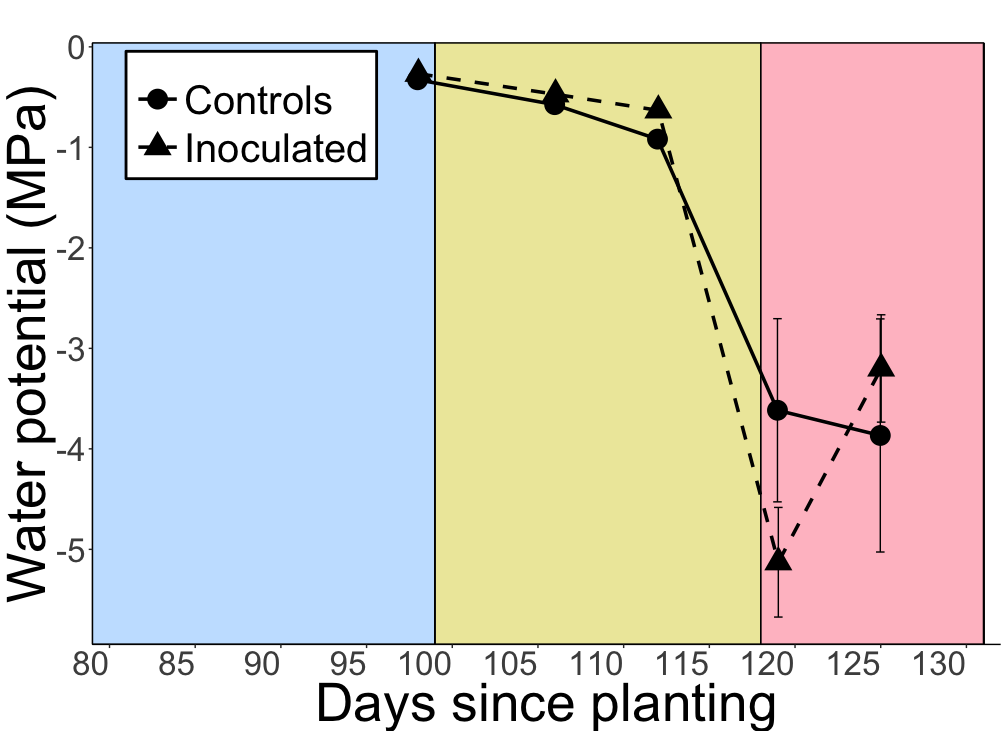


**Figure S1.** Predawn leaf water potential did not significantly differ between control and inoculated groups at P≤0.05. All values are expressed as means ± SE. The blue background color indicates the well-watered period, yellow indicates the moderate drought period before soil moisture declined to zero (days 99-117), and pink indicates the severe drought period after soil moisture declined to zero (days 118-131).


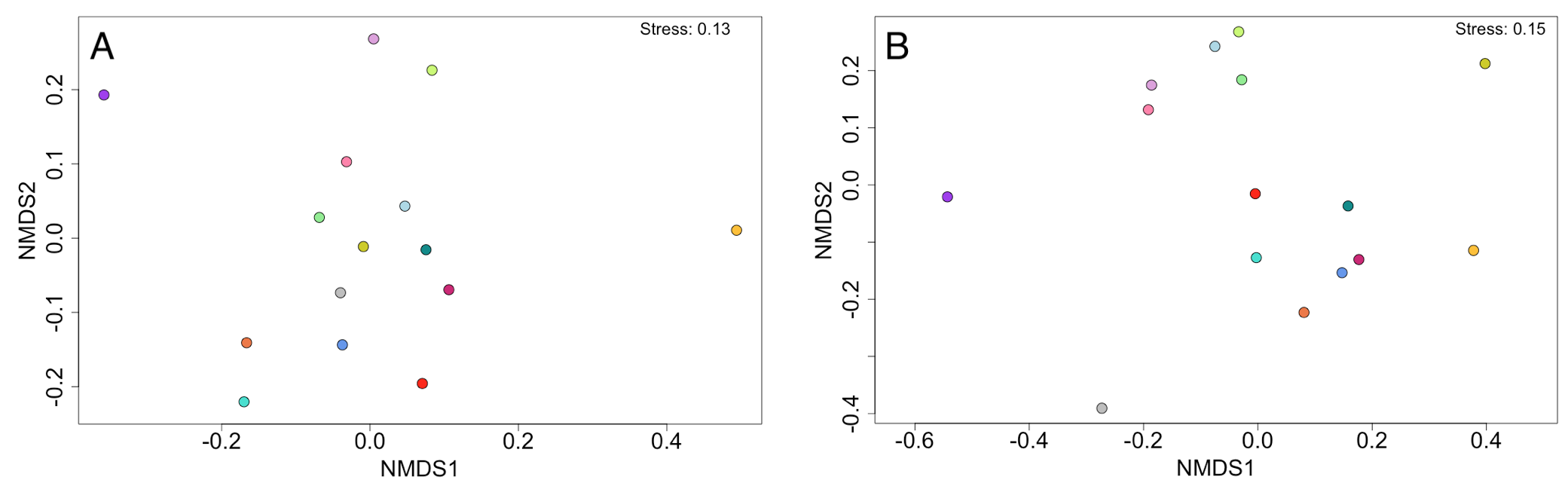


**Figure S2.** Non-metric multidimensional scaling (NMDS) plots of bacteria (A) and fungi (B) demonstrating differences in the composition of the 15 soil inocula applied to plants in the inoculated group (using homogeneity of multivariate dispersions, average distance to the median was 0.4781 for bacteria and 0.6038 for fungi). Control plants in sterile sand were not inoculated.
